# Investigation of an anthrax outbreak in Makoni District, Zimbabwe

**DOI:** 10.1186/s12889-021-10275-0

**Published:** 2021-02-05

**Authors:** Richard Makurumidze, Notion Tafara Gombe, Tapuwa Magure, Mufuta Tshimanga

**Affiliations:** 1 Department of Community Medicine, College of Health Sciences, University of Zimbabwe, Harare, Zimbabwe; 2 National AIDS Council, Harare, Zimbabwe

**Keywords:** Zimbabwe, Makoni, Outbreak, Anthrax, *Bacillus anthracis*

## Abstract

**Background:**

Anthrax continues to be a disease of public health importance in Zimbabwe, with sporadic outbreaks reported annually in many parts of the country. A human anthrax outbreak occurred in wards 22 and 23 of Makoni District from mid-June 2013 to end of January 2014, following cattle deaths in the wards. Laboratory tests confirmed anthrax as the cause for the cattle deaths. This study investigated the clinical characteristics, distribution of cases (places, person and time) and risk factors for contracting the anthrax disease. We also assessed the environment, district preparedness and response, and outbreak prevention and control measures.

**Methods:**

We conducted an outbreak investigation using a mixed-methods design. A 1:1 case-control study was used to assess risk factors for contracting anthrax. The controls were frequency matched to cases by sex. Data were collected using a structured interviewer-administered questionnaire. Environmental assessment, district preparedness and response, and outbreak prevention and control measures were assessed using a checklist, observations, and key informant interviews. Multivariable unconditional logic regression analysis was performed to identify independent risk factors associated with contracting anthrax.

**Results:**

We interviewed 37 of the 64 cases, along with 37 controls. All the cases had cutaneous anthrax, with the hand being the most common site of the eschar (43%). Most of the cases (89%) were managed according to the national guidelines. Multivariable analysis demonstrated that meat sourced from other villages [vs butchery, OR = 15.21, 95% CI (2.32–99.81)], skinning [OR = 4.32, 95% CI (1.25–14.94)], and belonging to religions that permit eating meat from cattle killed due to unknown causes or butchered after unobserved death [OR = 6.12, 95% CI (1.28–29.37)] were associated with contracting anthrax. The poor availability of resources in the district caused a delayed response to the outbreak.

**Conclusion:**

The described anthrax outbreak was caused due to contact with infected cattle meat. Although the outbreak was eventually controlled through cattle vaccination and health education and awareness campaigns, the response of the district office was initially delayed and insufficient. The district should strengthen its emergency preparedness and response capacity, revive zoonotic committees, conduct awareness campaigns and improve surveillance, especially during outbreak seasons.

**Supplementary Information:**

The online version contains supplementary material available at 10.1186/s12889-021-10275-0.

## Background

Anthrax is a bacterial infection caused by *Bacillus anthracis*, a Gram-positive, spore-forming, rod-shaped bacterium [1]. Globally, approximately 2000–20,000 human cases of anthrax occur each year [2]. Anthrax in humans is often a result of contact with infected meat from livestock and wildlife [1]. The most common form of anthrax in humans is cutaneous, while inhalation and gastrointestinal forms are less frequent [1]. During the past three decades, there has been a progressive global reduction in the number of reported cases of anthrax in livestock, and this might be attributed to the efforts made by national programs [2]. Anthrax is still endemic in most African countries, majority of which experience at least one human outbreak per year [3]. South Africa, generally reports less than five and occasionally zero human outbreaks annually, despite the continued occurrence of the disease in wildlife in the various parks [4]. A study in 2018 reported that, in spite of effective control programs in Botswana, Zimbabwe, and Zambia, the disease remained endemic in at least the latter two countries [4].

Anthrax continues to be a disease of public health importance in Zimbabwe since sporadic outbreaks are reported annually in many parts of the country [5, 6]. The outbreaks usually occur during the dry (July to October) and early to mid-wet summer (November to February) periods [7]. The disease was first diagnosed in the country in 1898 in the Matabeleland region. The largest recorded outbreak in humans and possibly the largest among animals occurred in 1978–1980 during the peak of the liberation war. The disease spread over time from area to area, until six of the eight provinces were affected, and over 10,000 human cases and 182 human deaths were documented. The number of human anthrax cases recorded during this period was far more than the previous years, during which usually less than a dozen cases were recorded annually [8–10]. Since then, sporadic outbreaks have been reported regularly in many parts of the country. The has been further exacerbated by the current deterioration of the economic environment in the country, which has led to poor implementation of veterinary prevention and control measures.

Human anthrax cases were reported to have started in mid-June, 2013 in wards 22 and 23 of Makoni District. The District Veterinary Department also reported cattle deaths in the same area during this period. We have investigated this human anthrax outbreak and reported the clinical characteristics, distribution of anthrax cases (place person and time), risk factors for contracting the disease, environmental assessment, district preparedness and response, and outbreak prevention and control measures.

## Methods

### Study setting

The study was conducted in Manicaland Province, Makoni District, Wards 22 and 23 in Zimbabwe [11]. Anthrax is endemic in Manicaland Province of Zimbabwe and cases in cattle and humans are annually reported. Thirty-seven human anthrax cases were recorded in 2011, and 49 human anthrax cases were recorded in 2012. The cases recorded in 2012 were reported from Buhera, Chipinge, Mutare, and Mutasa districts in the province [12].

### Study design

We conducted an outbreak investigation using a mixed-methods design. A 1:1 case-control study was used to assess outbreak risk factors for contracting anthrax, while data from key informants was collected qualitatively. The controls were frequency matched to cases by sex.

### Definition of cases and controls

A case was defined as any person who resided in wards 22 and 23 of Makoni District where laboratory diagnosis of anthrax in cattle was confirmed, who presented with itching in an affected area, followed by the appearance of a painful lesion, followed by the formation of papules, which then vesiculated and eventually developed into a depressed black eschar, between the 19th of June 2013 and the 29th of January 2014. A control was defined as any person who resided in wards 22 and 23 of Makoni District where laboratory diagnosis of anthrax was confirmed, who did not develop any disease with similar symptoms during the specified period.

### Identification of cases and controls

The cases were identified from the line list that was available at Makoni Rural Hospital, which services the two wards and through active case finding in the community. Since data from most of the cases were collected retrospectively, we collected the addresses (village, ward, headmen, nearest school) from the hospital line list and contacted the cases in the community for data collection. Active case finding was through snow-balling and a community outreach clinic conducted by us. The new cases identified were added to the outbreak line list. Individuals diagnosed with chickenpox, skin reactions caused by drugs, acute skin diseases or other diseases that vaguely mimic anthrax were evaluated by a clinician, who was part of the team to determine inclusion or exclusion of the case in the study. The controls for the study were selected from the neighbourhood of the cases. The nearest homestead in any direction from the household of a particular case was approached for a control belonging to the same sex. Unavailability of a control at the homestead, however, resulted in a repetition of the same process until a control was found.

### Sample size

A minimum sample size of 66 (33 controls and 33 cases) was required assuming 95% two-sided confidence interval, 80% power, 59.7% of controls exposed, 91.94% of cases exposed, and the least extreme odds ratio to be detected being 7.7 [13].

### Data collection

Data were collected using an interviewer-administered questionnaire (Additional file 1). The questionnaire collected data related to demography, medical history, risk factors for contracting the disease, and awareness about anthrax. Outpatient medical records were reviewed to get extra medical information on history, including the presenting symptoms, physical examination (signs), and management of the cases. An Integrated Disease Surveillance Response (IDSR) checklist was used to assess the emergency preparedness response of the district [14]. An environmental health assessment was done to assess the processes and rituals involved in the burial of carcasses, availability of dip tanks, and adequacy of grazing land. Key informant interviews were conducted with the District Medical Officer, District Veterinary Officer, District Environmental Health Officer, and the Environmental Health Technicians (EHTs) at Makoni Rural Hospital.

### Data analysis

The data were analyzed using Stata 16 [15]. The categorical baseline characteristics of the cases and controls were compared using frequencies, proportions, and Pearson’s Chi-square test. The median age was calculated for both cases and controls, and the Wilcoxon rank-sum test was used to determine any difference that may be present. We performed unconditional univariable and multivariable logistic regression to identify factors associated with contracting anthrax. The sex variable used for frequency matching was included in the regression model. We assessed for collinearity by calculating the variance inflation factor (VIF) with a cut-off value of 10, and found that there was none among the exposure variables. All variables associated with *p*-value < 0.1 in the univariable analyses were included in the multivariable model and stepwise backward elimination was performed for multivariable regression. We calculated the Bayesian information criterion (BIC) at every step, and chose the model which had the least BIC as our final model.

### Anthrax in animals - diagnosis and case definition

The recommended laboratory diagnostic procedure in Zimbabwe, for anthrax in animals is a blood smear obtained from a nipped ear or staining and microscopic examination of peripheral blood fixed with alcohol. Microscopic examination using the Giemsa stain shows purple-stained bacilli with red capsules, while Gram stain shows the typical square-ended gram-positive rods [7]. Although the defining characteristic of anthrax in animals is sudden death, which may be accompanied by bleeding from the orifices [1], the clinical diagnosis in animals was suspected if an animal presented with any one of the following signs and symptoms; cessation of feeding and drinking, staggering and falling, tremors, convulsions, massive oedema, swollen lymph nodes in the neck region, difficulty in breathing, bleeding from all orifices due to blood clotting problems, or sudden death [7].

## Results

### Anthrax in animals

The findings from the field investigation showed that the first cattle death occurred on the 12th of June, 2013. Sporadic cattle deaths then occurred from June 2013 through October 2013, but the situation worsened towards the end of November 2013. Most of the cattle deaths occurred in December 2013. The Veterinary Department collected specimens from a few cattle that were dying and sent them to the Provincial Veterinary Laboratory on the 24th of December 2013, where a laboratory diagnosis of anthrax was confirmed. The anthrax diagnosis in the rest of the animals was clinical. An estimated 180 cattle died in the wards 22 and 23. The exact number could not be ascertained since the Veterinary Department was not on the ground. Deaths of animals other than cattle, such as goats, pigs, and chicken were also reported, but the actual numbers could not be ascertained.

### Anthrax in humans

#### Study participants

We identified 64 cases in the wards 22 and 23 through active surveillance and from the hospital line list. All the cases were “probable” based on their clinical characteristics and epi-linkage to animal cases, that were laboratory confirmed. We managed to reach and interview 37 of the 64 cases.

#### Baseline characteristics of cases and controls

We interviewed 37 cases and 37 controls. The median age in years for cases and controls was 34 (interquartile range-IQR: 22–42) and 28 (IQR: 25–46), respectively. Males constituted 73% (27) of cases and 70.3% (26) of controls. While most of the cases (67.6%) and controls (59.5%) had secondary education and above, most of the cases and controls were not employed (59.5%). Generally, the baseline characteristics for cases and controls were comparable (Table 1).

**Table 1:** Baseline characteristics of cases and controls from the anthrax outbreak in 2014

Characteristic	Categories	Controls	Cases	***p***-value (χ2)
Sex	Female	11 (29.7)	10 (27.0)	0.797
	Male	26 (70.3)	27 (73.0)	
Median Age (IQR)		28 (25–46)	34 (22–42)	0.998^b^
Marital Status	Has partner	15 (40.5)	22 (59.5)	0.104
	No partner	22 (59.5)	15 (40.5)	
Education	Primary and below	15 (40.5)	12 (32.4)	0.469
	Secondary and above	22 (59.5)	25 (67.6)	
Employment	Employed	5 (13.5)	9 (24.3)	0.343
	Peasant farmer	10 (27.0)	6 (16.2)	
	Unemployed	22 (59.5)	22 (59.5)	
Religion	^a^ Traditional churches	18 (48.7)	19 (5.4)	0.330
	Apostolic	13 (38.1)	8 (21.6)	
	Others	6 (16.2)	10 (27.0)	

^a^ Traditional churches – Anglican, Methodist, Roman Catholic and Reformed Church of Zimbabwe

^b^Wilcoxon rank-sum, *IQR* Interquartile range

#### Case fatality rate

One community death suspected to have been due to anthrax disease was reported. The individual did not report to a health facility, but according to witnesses, he developed an eschar on the chest wall, followed by swelling of the left arm, and shortness of breath. The victim was known to have been involved in the skinning of cattle and consumed roasted meat during skinning. He died at the age of 40 years on the 13th of January, 2014. The outbreak thus had an estimated case fatality rate of 0.016 (1/64).

#### Clinical characteristics

**Fig. 1:**
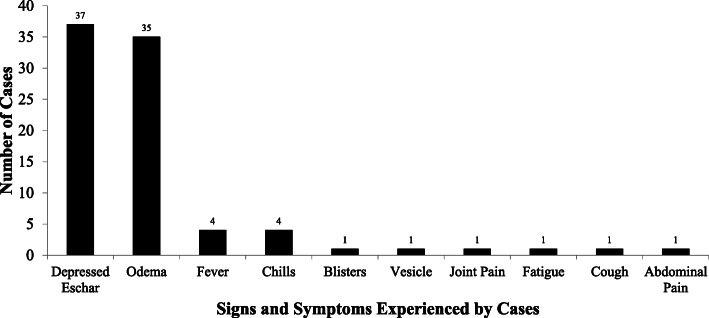
Symptoms and signs experienced by the cases during the anthrax outbreak in 2014

**Fig. 2:**
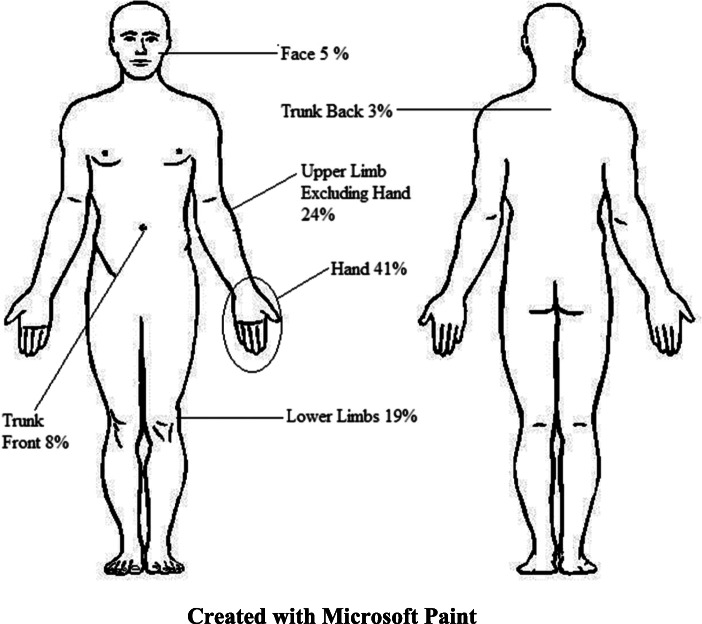
Sites of the depressed eschars in the cases during the anthrax outbreak in 2014 (Created by the authors with Microsoft Paint)

None of the 37 cases reported history of anthrax disease before. All the 37 cases presented with a depressed eschar. The other signs and symptoms experienced are summarized in Fig. 1. Most of the depressed eschars affected the hands (41%), while the back of the trunk was the least affected (Fig. 2). Three of the interviewed cases were admitted to a hospital, where they spent 2, 5, and 12 days, respectively. Laboratory tests, including the one for the diagnosis of anthrax were not conducted for any of the cases, that presented to the health facilities. Thirty-four cases (89%) were managed according to national guidelines on the management of anthrax (Doxycycline for mild cases and Benzyl or Procaine Penicillin for severe cases) [7]. Eleven of the cases self-treated with traditional herbs such as *chikohwa, muzeze, changamire, gakakava, chimutara* and leaves of gumtree before presenting to the health facilities.

#### Distribution of cases - place

**Fig. 3:**
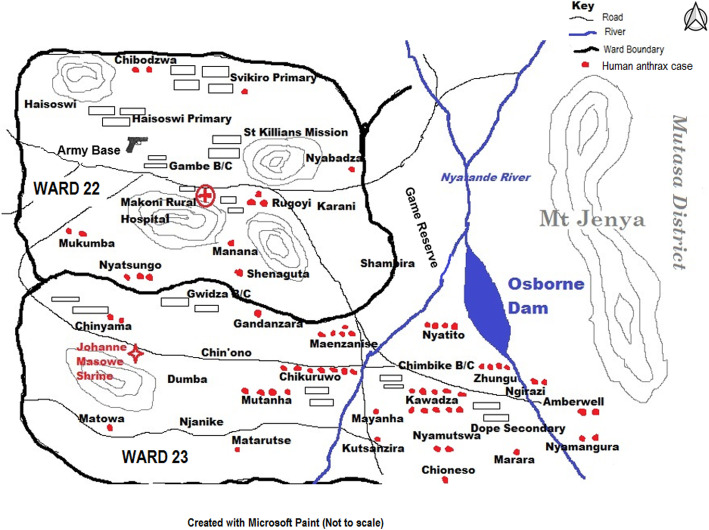
The spot map of the human anthrax cases during the outbreak in 2014 (Created by the authors with Microsoft Paint)

The anthrax outbreak affected the wards 22 and 23 of Makoni District. The two wards had 64 cases of anthrax reported in total, and ward 23 was the worst affected, with 78% (50) of the total cases. Of the 37 cases interviewed, 28 were from ward 23 and 9 were from ward 22. Twenty-six villages were affected in the two wards. Figure 3 shows the spot map of the outbreak.

#### Distribution of cases - time

**Fig. 4:**
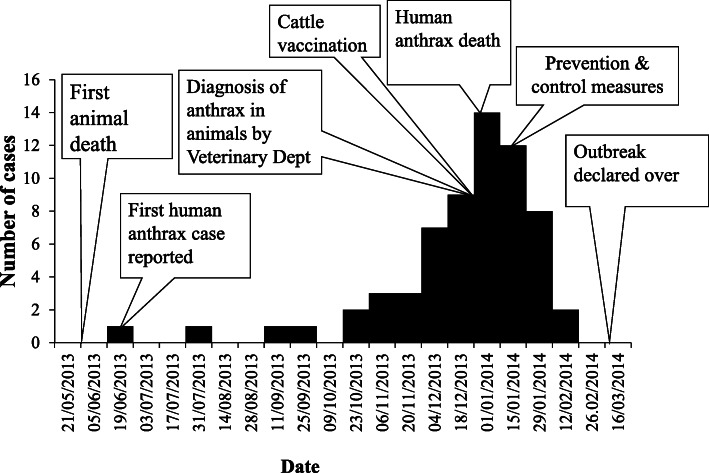
Epidemiological curve for the anthrax outbreak in Makoni District Ward 22 and 23 in 2014

The first probable human anthrax case was reported on the 19th of June, 2013. This patient case sought medical attention at Makoni Rural Hospital and was referred to Rusape District Hospital, which further referred the case to Mutare Provincial Hospital, where a clinical diagnosis of cutaneous anthrax was made, and the patient was treated empirically. The human anthrax cases started to increase rapidly in December 2013, peaked in mid-January 2014, and finished by the 29th of January 2014. These field findings were contrary to those reported by the District Veterinary Office and District Medical Office. The District Veterinary Office reported that the first cattle death occurred on the 6th of December, 2013 and the District Medical Office reported that the first case of human anthrax was reported on the 21st of December, 2013. Figure 4 shows the epidemiological curve of the outbreak.

#### Risk factors for contracting anthrax

Multivariable analysis demonstrated that sourcing cattle meat from other villages [vs butchery, OR = 15.21, 95% CI (2.32–99.81)], skinning cattle [OR = 4.32, 95% CI (1.25–14.94)], and belonging to a religion that permits eating meat from cattle slaughtered with undiagnosed illness or butchered after an unobserved death [OR = 6.12, 95% CI (1.28–29.37)] were associated with contracting anthrax during the outbreak (Table 2).

**Table 2:** Bivariate and multivariable logistic regression for factors associated with contracting anthrax in 2014

Variables	Categories	Controls	Cases	OR	95% CI	***p***-value	aOR	95% Cl
Sex	Female	11 (29.7)	10 (27.0)	1			1.14	0.42–3.14
	Male	26 (70.3)	27 (73.0)	1.14	0.42–3.14	0.797		
Marital Status	Has partner	15 (40.5)	22 (59.5)	1				
	No partner	22 (59.5)	15 (40.5)	0.46	0.18–1.18	0.106		
Education	Primary and below	15 (40.5)	12 (32.4)	1				
	Secondary and above	22 (59.5)	25 (67.6)	1.42	0.55–3.68	0.469		
Employment	Employed	5 (13.5)	9 (24.3)	1				
	Peasant farmer	10 (27.0)	6 (16.2)	0.33	0.07–1.48	0.352		
	Unemployed	22 (59.5)	22 (59.5)	0.56	0.16–1.93			
Religion	^a^ Traditional churches	18 (48.7)	19 (5.4)	1				
	Apostolic	13 (38.1)	8 (21.6)	0.58	0.20–1.74	0.337		
	Others	6 (16.2)	10 (27.0)	1.58	0.48–5.24			
^b^ Ate meat	No	17 (46.0)	4 (10.8)	1				
	Yes	20 (54.0)	33 (89.2)	**7.00**	**2.06–23.82**	**0.002**		
Source of meat	Butchery	11 (29.7)	2 (5.4)	1				
	Other villages	10 (27.0)	27 (73.0)	**14.85**	**2.79–79.06**	**0.001**	**15.21**	**2.32–99.81**
	Own cattle	6 (16.2)	6 (16.2)	5.5	0.84–36.20		7.25	0.84–62.57
	Missing	10 (27.1)	2 (5.4)	–	–	–	–	–
^c^ Cutting meat	No	20 (54.1)	7 (18.9)	1				
	Yes	17 (45.9)	30 (81.1)	**5.32**	**1.91–14.77**	**0.002**		
Skinning	No	29 (78.4)	15 (40.5)	1				
	Yes	8 (21.6)	22 (59.5)	**5.04**	**1.77–14.36**	**0.001**	**4.32**	**1.25–14.94**
Cooking	No	23 (62.2)	12 (32.4)	1				
	Yes	14 (37.8)	25 (67.6)	**3.42**	**1.32–8.91**	**0.012**		
^d^ Cuts	No	31 (83.8)	22 (59.5)	1				
	Yes	6 (16.2)	15 (40.5)	**3.50**	**1.18–10.51**	**0.024**		
Hide preparation	No	26 (70.3)	21 (56.8)	1				
	Yes	11 (29.7)	16 (43.2)	1.80	0.69–4.70	0.229		
Heard of anthrax before	No	10 (27.0)	19 (51.4)	1				
	Yes	27 (77.0)	18 (48.6)	**0.35**	**0.13–0.93**	**0.034**		
^e^ Religion permissions	No	33 (89.2)	21 (56.8)	1				
	Yes	4 (10.8)	16 (43.2)	**6.29**	**1.85–21.39**	**0.003**	**6.12**	**1.28–29.37**

*OR* odds ratio, *aOR* adjusted odds ratio, *CI* confidence interval

^a^Traditional churches – Anglican, Methodist, Roman Catholic and Reformed Church of Zimbabwe

^b^Ate meat from a cattle slaughtered due to unknown illness or butchered after an unobserved death

^c^Cutting skinned meat prior to cooking

^d^Cut him- or herself while skinning or cutting meat

^e^ Religion which permits eating meat from cattle killed due to unknown causes or butchered after an unobserved death

### Environmental assessment

Most of the cattle that died were buried unsupervised and inappropriately. Animal carcasses were found in open spaces, which allowed dogs and vultures to consume them. Reports of people throwing infected meat in the nearby Osborne Dam also emerged. There was a game reserve in ward 22 and close to Osborne Dam that bordered some of the affected villages. Cattle from the surrounding communities came together and grazed in the same areas with the game animals since the game reserve fence was not intact. Both wards 22 and 23 were found to have inadequate grazing land and pastures, with most of the grazing area comprising of short grass. Ward 23 bordered Mutasa District, which had experienced an anthrax outbreak in the previous year and cattle from the two districts shared grazing land.

### District preparedness and response

The rural hospital that services the two wards had an adequate stock of drugs (Doxycycline for mild cases and Benzyl or Procaine Penicillin for severe cases), which were used to treat anthrax during the outbreak period. This was assessed using the stock cards as of the 31st of November, 2013. The EHTs on the field had no modes of transport and personal protective equipment (PPE), i.e. overalls/work suits, gumboots, and heavy-duty gloves to use during the outbreak. The EHTs were also not provided with allowances. Information, education, and communication materials were not available during the early stages of the outbreak response and only became available later. The district had no emergency preparedness and response plan, and the zoonotic committees were not functional. The cumulative period of the outbreak was 7 months, but a concrete response started only after 6 months despite the first case having been reported in June 2013. The line list of cases was also found to be incomplete.

### Outbreak prevention and control measures

The district started concrete outbreak control measures on the 21st of January, 2014. The team dispatched to institute the outbreak control measures comprised of two EHTs, one veterinary officer, and a public health officer from the University of Zimbabwe, Field Epidemiology Training Programme. Health education was offered at 15 of the 16 primary and secondary schools in both wards. A meeting was organized through the chief of the area with village heads of the two wards. Of the 53 village heads, 14 managed to attend the meeting, where health education, advocacy, and lobbying to control the outbreak were discussed. A total of 5896 people were reached with health education in both wards. Active case finding was conducted in the community, and a total of 8 more cases were identified. An outreach clinic to treat new cases and review old cases was established at Dope Secondary School in ward 22.

Lime was applied to areas where cattle had died. The team also assisted in the supervised burial of new cattle deaths and reburial of carcasses that had been disposed inappropriately. Two butcheries in the wards were barred from selling meat during the outbreak period. Slaughtering of cattle was stopped in the butcheries and in the surrounding communities. Dried meat was confiscated from the villages. The amount of meat confiscated and destroyed could not be ascertained since there was no scale to weigh the meat. The Veterinary Department vaccinated the cattle against anthrax in the wards. The registered number of cattle at the dip tanks of the three wards was about 5000, of which 4000 were vaccinated (80%). The department also stopped issuing of permits for cattle movement during the period.

## Discussion

The described anthrax outbreak in Zimbabwe affected cattle and caused transmission to humans. Most of the cases of human anthrax were cutaneous with the hands being the most affected, and the case fatality rate was low. The outbreak was prolonged, and it took time for the district to institute control measures.

The anthrax eschar was more commonly found on the hands than other body parts, a finding that was consistent with other studies [13, 16]. Since hands are used for handling meat, they are at higher risk of developing abrasions, bruises, and cuts, thereby creating a route of entry for the anthrax spores. The case fatality rate for this outbreak was very low, another finding that was consistent with other studies that had recorded low fatality cases in anthrax outbreaks [17, 18]. The low case fatality rate in anthrax outbreaks might be attributed to the fact that the most common form of anthrax, i.e. cutaneous anthrax is known to have the least mortality rate as compared to other forms of anthrax [1]. All the interviewed cases in our study had cutaneous anthrax, while the victim who died in the outbreak might have developed either gastrointestinal or respiratory anthrax, both of which have higher mortality as compared to cutaneous anthrax [19, 20].

The following were found to be risk factors for contracting anthrax; sourcing meat from other villages, skinning, and belonging to religions permit eating meat from cattle killed due to unknown causes or butchered after an unobserved death. These findings are consistent with other studies done locally and might be due to the similarities in the practices [13, 21, 22]. Since the skinning process increases the probability of developing cuts and abrasions, which can create access routes for the spores to the sub-dermal tissue, skinning is considered as a risk factor for contracting anthrax [1]. Those belonging to religions that permit eating meat from animals killed due to unknown causes or butchered after an unobserved death were found to be at risk of contracting anthrax. This finding, however, is not consistent with another local study where one’s religious belief related to the consumption of meat from cattle killed due to unknown causes or butchered after an unobserved death was not associated with contracting anthrax [13, 21].

The environment assessment indicated some risk factors associated with anthrax contraction among animals. The outbreak started a few months before the rainy season, a period typically associated with a lack of grazing grass, resulting in an inadequacy of grazing land and pastures, in addition to the available grass being short, all of which predispose grazing cattle to ingestion of the anthrax bacilli due to overgrazing [23, 24]. Anthrax spores can survive for an extended period if the soil conditions are conducive [25]. One of the wards also bordered a game reserve, and sharing of grazing land with game animals has been implicated in the transmission of anthrax to livestock [26].

The outbreak was prolonged, and it took time for the district to start instituting outbreak control measures since the district did not have an emergency preparedness and response plan in addition to unavailability of adequate resources such as PPE. However, despite the delay in instituting control measures, the outbreak was brought under control within 2 months of laboratory confirmation of the first animal case. The district used chloride of lime to disinfect sites where animal carcasses had died. However, this practice is no longer recommended since chloride of lime is rapidly neutralized by organic matter. Moreover, chloride of lime is corrosive, carcinogenic, unstable, and potentially explosive, which makes its use without proper PPE dangerous [1, 3]. The ability of the resulting calcium residue to actively encourage spore preservation also results in ineffective destruction of anthrax spores [27]. The recommended approach presently in use is to bury animal carcasses deep down in the ground. The zoonotic committees that are key in quick identification of zoonotic diseases were not functional at neither the district nor the local levels. The outbreak was curbed, as soon as the district started to institute outbreak control measures. The massive health education and awareness campaigns conducted could have also significantly contributed to curbing the outbreak [17, 21]. Studies have shown that awareness and knowledge about the modes of transmission of anthrax, signs and symptoms, and preventive measures among community members reduce exposure to risk factors [28].

### Strength of the study

Our study practically implemented most of the components of an outbreak investigation. A team was set up and prepared for the fieldwork associated with the outbreak. We established a case definition that was used to identify, count, and create a line list of cases. We described the outbreak in terms of person, place, and time together with risk factor analysis. Outbreak control and prevention measures were instituted, results disseminated, and later the outbreak was successfully controlled.

### Limitations

However, our outbreak investigation had limitations. There was no laboratory diagnosis of anthrax in humans, although laboratory confirmation was obtained for anthrax in a few animals. This was done in accordance with the Zimbabwe guidelines, which state that collection of humans specimens may not be necessary if diagnosis of anthrax has already been confirmed in animals from the area of the outbreak. The collection of specimens is also not encouraged owing to the risk of contamination and transmission of the anthrax bacilli in case of unsupervised collection and improper handling of specimens. The failure to perform laboratory diagnosis in humans might have resulted in misclassification, particularly of non-cases, i.e. patients with other skin lesions, thus affecting the strength of our measures of association. Of the 64 cases recorded in wards 22 and 23, we only managed to interview 39, while the other cases could not be interviewed due to issues associated with distance and feasibility. We also did not compare the demographic characteristics of the interviewed cases with the other cases to assess similarity and possible bias of our sample. The small sample size may have affected the precision of our point estimates. Recall bias could also have affected our results since data were collected after exposure and cases are usually more likely to remember the incidences of exposures more than controls. We did not collect information from non-responding controls, whose characteristics could have influenced our results.

## Conclusion

The described outbreak had a low case fatality rate, and most of the human anthrax cases were cutaneous. We found sourcing meat from other villages, skinning, and belonging to religions which permit eating meat from cattle killed due to unknown causes or butchered after an unobserved death to be risk factors for contracting anthrax. The district was not prepared for handling the outbreak, and thus delayed the institution of appropriate prevention and control measures. Zoonotic committees were not functional, and the coordination between the health and veterinary departments during the outbreak response was weak and inadequate. We recommended the following based on our investigation findings: strengthening of the district capacity and training of health workers in epidemic preparedness and response, improvement in the surveillance efforts for anthrax during the high-risk period, providing the EHTs with motorcycles, health education on anthrax in the community during the high-risk period, and activation of zoonotic committees with complete participation of the veterinary department.

## Supplementary Information

**Additional file 1.** Data collection tools: Investigation of an anthrax outbreak in Makoni District, Zimbabwe.

## Data Availability

The data which were used for this outbreak investigation report are not available on the public domain, but anyone interested in using the data for scientific purpose is free to request permission from the corresponding author: Dr. Richard Makurumidze**,** University of Zimbabwe College of Health Sciences, Department of Community Medicine**.** Email: richardmakurumidze@gmail.com.

## Abbreviations

CI: confidence interval
OR: odds ratio
EHT: Environmental Health Technician
PPE: personal protective equipment
