# Supplementary material for: Investigation of an anthrax outbreak in Makoni District, Zimbabwe
Source: BMC Public Health. 2021 Feb 5;21:298. doi: 10.1186/s12889-021-10275-0 (PMC7866675; doi:10.1186/s12889-021-10275-0)
Supplement: Supplementary file 1 — Additional file 1. Data collection tools: Investigation of an anthrax outbreak in Makoni District, Zimbabwe. [file 12889_2021_10275_MOESM1_ESM.docx]

**DATA COLLECTION TOOLS: INVESTIGATION OF AN ANTHRAX OUTBREAK IN MAKONI DISTRICT, ZIMBABWE**

**PART 1: QUESTIONNAIRE**

Status of Respondent Status **Case**  **Control** Sequence Number……….

Date Questionnaire was administered (DD/MM/YYYY) ----/----/--------

**1. Demographic Data**

1. District ………………Ward ……………………Village…………………….

2. What was your age on you last birthday? ………… Date of Birth ………………………

Sex Male Female

3. What is your level of education? Never Been to School Primary 2 Year Secondary 4 Year Secondary Tertiary/College

4. What is your marital status? Never Married Married Divorced Separated Widowed

5. How many are you in your household? . ............................

6. What is your employment status? Not Employed Full time Employment

Part time Employment Self Employed Peasant Farmer Student

7. What is average monthly income? None < $100 Between $100 and $200

Between $200 and $300 Between $300 and $400 Between $300 and $400 >$500

8. What is your religion? Apostolic SDA AFM Roman Catholic Methodist Traditional Other

9. Does your religion permits eating of animals that has been slaughtered due to ill health or died on its own? Yes No

**2. Clinical Information**

10. What was the date of onset of symptoms? (DD/MM/YYYY) ----/----/-------- (*Verify with medical records)*

11. What was the date of diagnosis at health institution? (DD/MM/YYYY) ----/----/-------- (*Verify with medical records)*

12. Have you ever had anthrax before?

No Once Twice Three times and above

**Signs and Symptoms**

13. What are the signs and symptoms did you experience during your illness with anthrax?

*(Tick the symptoms and signs in the tables below and also verify some of them with the medical records)*

**14. General**

|  | Yes | No | Unknown |
| --- | --- | --- | --- |
| Fever |  |  |  |
| Chills |  |  |  |
| Myalgia |  |  |  |
| Joint Pain |  |  |  |
| Fatigue |  |  |  |
| Other, specify |  |  |  |

**15. Cutaneous**

|  | Yes | No | Unknown |
| --- | --- | --- | --- |
| Papule |  |  |  |
| Vesicle |  |  |  |
| Depressed eschar |  |  |  |
| Oedema around eschar |  |  |  |
| Erythema |  |  |  |
| Blisters |  |  |  |
| Other, specify |  |  |  |

16. If Yes to any of the Cutaneous symptoms and signs state the site

|  | Lower Limbs | Upper Limbs | Trunk Back | Trunk  Front | Face | Head |
| --- | --- | --- | --- | --- | --- | --- |
| Papule |  |  |  |  |  |  |
| Vesicle |  |  |  |  |  |  |
| Depressed eschar |  |  |  |  |  |  |
| Erythema |  |  |  |  |  |  |
| Blisters |  |  |  |  |  |  |

**17. Pulmonary**

|  | Yes | No | Unknown |
| --- | --- | --- | --- |
| Cough |  |  |  |
| Short of Breath |  |  |  |
| Chest pain |  |  |  |
| Other, specify |  |  |  |

**18. Gastrointestinal**

|  | Yes | No | Unknown |
| --- | --- | --- | --- |
| Nausea |  |  |  |
| Vomiting |  |  |  |
| Abdominal Pain |  |  |  |
| Diarrhoea |  |  |  |
| Other, Specify |  |  |  |

**19. Neurological**

|  | Yes | No | Unknown |
| --- | --- | --- | --- |
| Headache |  |  |  |
| Photophobia |  |  |  |
| Neck stiffness |  |  |  |
| Other, specify |  |  |  |

20. Did you seek medical attention? Yes No

21. If the client answer to 17 is Yes, where did you seek medical attention? Local Clinic Rural Hospital District Hospital Other, Specify…………

22. If you sought medical attention what is the name of the health institution? …………………

23. Before going to the health institution did you sought assistance somewhere else?

Traditional healer Prophet Pastor Self Medication Other, specify………..

24. Do you know of any traditional or local treatment of anthrax? Yes No

25. If you know of any traditional or local treatment, can you state it? ............................................

26. Were you admitted at the health institution? Yes No

27. If you were admitted what was:

Date of admission? ----/----/---- Date of Discharge? ----/----/------- Days in hospital? ………

28. Did you have any laboratory tests done to you? Yes No

*(If laboratory tests were done attach the tests and results on a separate sheet)*

29. The interviewer should check medication, treatment and medical procedures done to patient (*Verify with medical records)* ………………………………………………………………………………………………………………………………………………………………………………………………………………………………………………………………………………………………………

30. The interviewer should check if the management of the case was in line with national guidelines for management of anthrax? Yes No . If No state what was done wrong

…………………………………………………………………………………………………………………………………………………………………………………………………………………………………………………………………………………………………. ….

**Outcome**

31. Type of Anthrax Cutaneous Gastrointestinal Neurological Other, specify …………………………

32. Outcome of illness Complete Recovery Death Still recovering

33. If recovered duration of illness……………………………days

**3. Risk Factors for Contracting Anthrax**

34. Did you eat meat between 26 December 2013 and today? Yes No

35. What was your source of meat? Buying from villagers Receiving from villagers Butchery Other specify………………………………..

36. Did you ate meat from a dead animal? Yes No

37. Were you involved in Skinning meat of a dead animal? Yes No

38. Were you involved in cutting Yes No

39. Were you involved in cooking meat? Yes No

40. Did you handle a dead carcass or its products Yes No

41. Did you experience cuts or abrasion during skinning? Yes No

42. Did you experience cuts or abrasion during cutting meat? Yes No

43. Were you involved in the preparation of hide? Yes No

44. Do you think overcooking meat kills anthrax? Yes No

**History of Cattle Deaths**

|  | **Yes** | **No** | **If yes specify date or month** |
| --- | --- | --- | --- |
| 45. Have there been cattle deaths in this village? |  |  |  |
| 46. Have there been deaths of other animals not cattle in this village? |  |  |  |
| 47. Do you own cattle? |  |  |  |
| 48. Have any of your cattle died in the last one week or before. |  |  |  |
| 49. Did you slaughter your cattle due to ill-health in the last week or before? |  |  |  |
| 50. How many cattle died in your house hold? | //// | ///// |  |
| 60. Did you report to the Veteran Department? |  |  |  |

**If the household had an animal death or slaughtered animal due to ill-health ask the following**

61. What happened to the meat/carcass?

|  | **Yes** | **No** |
| --- | --- | --- |
| Destroyed |  |  |
| Sold |  |  |
| Dried as biltong |  |  |
| Others (specify) | | |

62. If the meat/carcass was destroyed were you involved in destroying meat/ carcass?

Yes No

63. If meat/carcass Destroyed describe how it was destroyed ………………………………………………………………………………………………………………………………………………………………………………………………………………………………………………………………………………………………………

64. What happened to the hide?

|  | **Yes** | **No** |
| --- | --- | --- |
| Destroyed |  |  |
| Dried for reuse as sitting or sleeping mattress |  |  |
| Dried for reuse as harnesses |  |  |
| Sold |  |  |
| Others (specify) | | |

65. If the hide was destroyed were you involved in destroying the hide? Yes No

67. If hide Destroyed describe how it was destroyed ………………………………………………………………………………………………………………………………………………………………………………………………………………………………………………………………………………………………………

**4. Knowledge About Anthrax**

68. Have you heard about anthrax before the problem of animal deaths in the community? Yes No

69. If yes state where? Radio Television Print Media Local Clinic

Other, specify……………………….. ..

70. What are the symptoms and signs of anthrax? Fever Eschar Nausea Abdominal Pain Diarrhoea Cough Other(s)…………………………………

71. How is anthrax prevented? Not eating meat from dead animals Vaccination of Animals Other(s) specify………………………………………………………………

**5. Practices on Anthrax**

In your opinion which practices predisposes human beings to getting anthrax? (Tick applicable boxes below).

|  | Yes | No |
| --- | --- | --- |
| 72. Handling carcass of animal that die on its own |  |  |
| 73. Skinning cattle that have died |  |  |
| 74. Buying/selling meat from animals that have died on their own. |  |  |
| 75. Trading with hides |  |  |
| 76. Killing an animal one thinks is going to die of illness |  |  |
| 77. Sharing or selling meat from dead animals with neighbors |  |  |
| 78. Regarding meat from animals which die on their own as cheap source of meat |  |  |
| 79. Not to burning carcasses of animals that have died on their own( Improper disposal) |  |  |
| Others (specify) | | |

Do you know of anyone in the village who has developed wounds on the face neck or arms after eating meat of the animals which died on its own? Yes No

If yes supply names of patients and village……………………………………

**Thank you for your time.**

**PART 2: ASSESSMENT OF THE ENVIRONMENT AND DISTRICT EMERGENCY PREPAREDNESS RESPONSE**

1. Sensitization meetings
2. Infection Control Issues
   1. Disinfectants
   2. Disposal of carcass
   3. Disposal of meat
3. Vaccination of Animals
4. Emergence Preparedness Response Plan availability
5. Evidence of Coordination (Availability of Zoonotic Committee)
6. Timelines
   1. 1^st^ case in community to arrival of 1^st^ case to Health Facility
   2. 1^st^ case at Health Facility & report to district
   3. Notification of District to concrete response
   4. Cumulative Period
7. Case Forms and Line List
8. Collecting and Processing of Laboratory Specimens
9. Outbreak Report
10. Final End of Outbreak Report
